# Na_2_Ga_7_: A Zintl–Wade Phase Related to “α-Tetragonal Boron”

**DOI:** 10.1021/acs.inorgchem.3c00790

**Published:** 2023-05-25

**Authors:** Chia-Chi Yu, Alim Ormeci, Igor Veremchuk, Xian-Juan Feng, Yurii Prots, Mitja Krnel, Primož Koželj, Marcus Schmidt, Ulrich Burkhardt, Bodo Böhme, Lev Akselrud, Michael Baitinger, Yuri Grin

**Affiliations:** † Max-Planck-Institut für Chemische Physik fester Stoffe, Nöthnitzer Str. 40, 01187 Dresden, Saxony, Germany; ‡ Helmholtz-Zentrum, Dresden-Rossendorf, Bautzener Landstraße 400, 01328 Dresden, Germany; § Jozef Stefan Institute, P.O. Box 3000, 1001 Ljubljana, Slovenia; ∥ Ivan Franko Lviv National University, Kyryla i Mefodia St. 57, 29005 Lviv, Ukraine

## Abstract

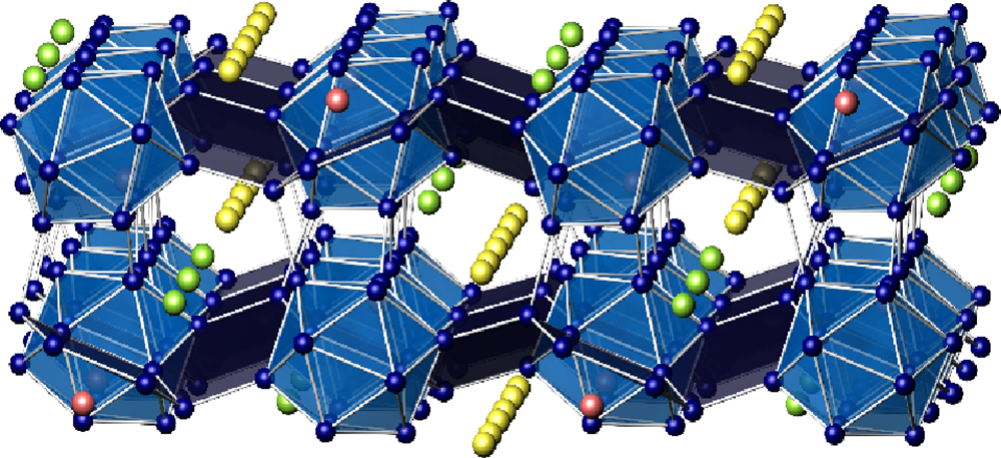

Na_2_Ga_7_ crystallizes with the orthorhombic space group *Pnma* (no. 62; *a* = 14.8580(6) Å, *b* = 8.6766(6) Å, and *c* = 11.6105(5) Å; *Z* = 8) and constitutes a filled variant of the Li_2_B_12_Si_2_ structure type. The crystal structure consists of a network of icosahedral Ga_12_ units with 12 exohedral bonds and four-bonded Ga atoms in which the Na atoms occupy the channels and cavities. The atomic arrangement is consistent with the Zintl [(4b)Ga]^−^ and Wade [(12b)Ga_12_]^2–^ electron counting approach. The compound forms peritectically from Na_7_Ga_13_ and the melt at 501 °C and does not show a homogeneity range. The band structure calculations predict semiconducting behavior consistent with the electron balance [Na^+^]_4_[(Ga_12_)^2–^][Ga^–^]_2_. Magnetic susceptibility measurements show that Na_2_Ga_7_ is diamagnetic.

## Abstract 2

Na_2_Ga_7_ is an electronically balanced line phase prepared from the elemental components or by redox reactions. The crystal structure is a filled variant Li_2_B_12_Si_2_ type of structure with a framework of [Ga_12_]^2−^ Wade and [Ga]^−^ Zintl anions hosting Na atoms in cavities and channels.

## 1 Introduction

Intermetallic compounds with high gallium content form a variety of complex crystal structures reflecting the difficulty of achieving a closed-shell configuration with three valence electrons.^1−8^ Within group 13 of the periodic table, gallium allotropes are structurally unique. Gallium transforms to an *fcc* packing like aluminum only at extreme conditions,^9,10^ and it has a much lower tendency than boron to form icosahedra, which have only been observed in δ-Ga.^11^ However, the topology of the α-Ga structure has been described by motifs related to open icosahedra.^12^ The crystal structures of alkali-metal gallides typically comprise Ga_12_ icosahedral units, which can be connected by four-bonded Ga atoms as in the cases of LiGa_2_,^13^ Li_3_Ga_14_,^14^ RbGa_7_,^15^ and CsGa_7_.^16^ The atomic connectivity in the mentioned compounds is thus reminiscent of the structure formerly considered as “α-tetragonal boron”^17−20^ or the Mg_2_B_24_C type.^21^

In the Na–Ga system, three compounds have been reported, showing different kinds of structural organization. In the gallium-rich side of the phase diagram, NaGa_4_^22^ is a representative of the BaAl_4_ type. The crystal structures of Na_7_Ga_13_^23^ and Na_22_Ga_39_,^24^ also reported earlier as Na_7_Ga_13_-II,^25^ comprise interconnected Ga_12_ and Ga_15_ clusters. The new equilibrium phase Na_2_Ga_7_ extends the series of binary phases with a Ga framework compound built up by [Ga_12_]^2–^ Wade and [Ga]^−^ Zintl anions. Na_2_Ga_7_ was first discovered by reacting Na_7_Ga_13_ with gaseous NH_3_, following previous research on the redox preparation of metastable cage compounds.^26−28^ Here, preparation methods, phase relations, crystal structures, and physical properties of Na_2_Ga_7_ are reported.

## 2 Experimental Section

### 2.1 Preparation of Na_2_Ga_7_

#### 2.1.1 Direct Reaction from the Elements

A total amount of 3 g of Na (Chempur, 99.9%) and Ga (Chempur, 99.99999%) with a molar ratio of Na:Ga = 2:7 were filled in a Ta ampoule under an Ar atmosphere and welded using an arc furnace. The ampoule was heated for 2 min at 1000 °C in an induction furnace and cooled to room temperature by removing it from the induction coil. Afterward, the ampoule was jacketed into a quartz tube under vacuum, annealed at 300 °C for 7 days in a tube furnace, and then quenched in water. The single-phase reaction product from PXRD consisted of crystalline metallic grains and was sensitive to air and moisture.

#### 2.1.2 Comproportionation of NaGa_4_ and Na_7_Ga_13_

A mixture of finely ground NaGa_4_ and Na_7_Ga_13_ in the molar ratio of 23:1 (eq 1) was pressed to a pellet (*d* = 10 mm; *h* = 2 mm). The pellet was sealed in a Ta ampoule under an Ar atmosphere and put in a tube furnace at 300 °C. After 1 h of annealing, Na_2_Ga_7_ was the main product from PXRD with residual NaGa_4_.
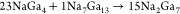
1

#### 2.1.3 Reduction of NaGa_4_

A mixture of finely ground NaGa_4_ and NaNH_2_ in the molar ratio of 7:1 (eqs 2 and 3) was pressed to a pellet (*d* = 10 mm; *h* = 2 mm). The pellet was placed in an open Ta crucible under an Ar atmosphere and annealed in a tube furnace at 300 °C. After 1 h, Na_2_Ga_7_ was the main product from PXRD with residual NaGa_4_. NaNH_2_ was not detected.
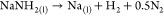
2
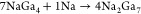
3

#### 2.1.4 Oxidation of Na_7_Ga_13_ with NH_3_

**Scheme 1:**
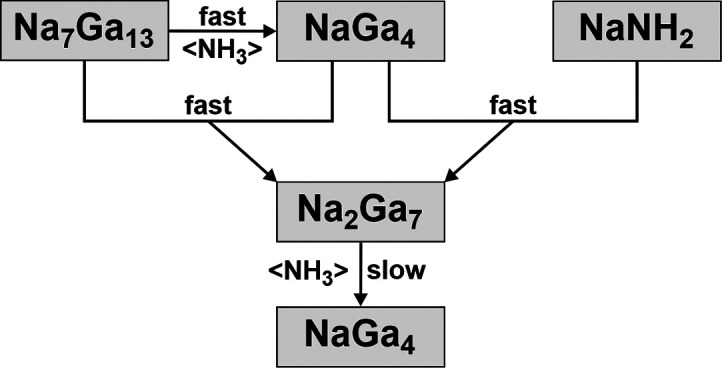
Reaction Products by Treating Na_7_Ga_13_ with NH_3_ at 300 °C

A DURAN glass crucible with approximately 50 mg of finely ground Na_7_Ga_13_ (*n* ≈ 0.05 mmol) was placed in a Schlenk glass reactor, which was connected to a pressure protection valve and an NH_3_/Ar gas supply. The reactor (*V* = 920 mL) was evacuated and refilled with gaseous NH_3_ (*p* ≈ 200 mbar; *n* ≈ 7.5 mmol) and then with Ar to atmospheric pressure. The loaded reactor was heated in a tube furnace at 300 °C for 2 h. After cooling, the system was evacuated and refilled with argon. Characterization of the products was conducted in an Ar-filled glovebox. The reaction product consisted of Na_2_Ga_7_ as the main and NaGa_4_ as the minority phase (Scheme 1). Amorphous contributions visible in PXRD patterns and sphere-shaped segregations in SEM images revealed the presence of elemental Ga (Figure S1). NaNH_2_ was not detected in PXRD.

#### 2.1.5 Thermal Decomposition of Na_7_Ga_13_

Fifty milligrams of finely ground powder of Na_7_Ga_13_ was filled in an open Ta crucible. The crucible was placed in a glass tube, which was evacuated to 10^–2^ mbar and closed. After heat treatment at 300 °C for 2 days, the product consisted mainly of Na_2_Ga_7_ with NaGa_4_ and residual Na_7_Ga_13_ as minority phases from PXRD (eqs 4 and 5).
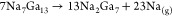
4
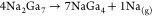
5

### 2.2 Oxidation of Na_7_Ga_13_ in Air

Fifty milligrams of finely ground powder of Na_7_Ga_13_ was filled in an Ar atmosphere in an open Al_2_O_3_ crucible. The crucible was placed in a Schlenck tube in a preheated tube furnace. Afterward, the vessel was opened to air. Oxidation experiments were performed at 300 and 800 °C. After 1 day at 300 °C, a gray powder was obtained, showing reflections from crystalline β-NaGaO_2_ and an amorphous background in PXRD. The gray component is assumed to be mainly elemental gallium from optical microscopy. After 1 day at 800 °C, the sample contained white particles of crystalline β-NaGaO_2_ and gray particles containing crystalline β-Ga_2_O_3_ and a residue of elemental Ga. The lattice parameters and diffraction intensities of β-NaGaO_2_ and β-Ga_2_O_3_ were consistent with the reported data (Table S1).^29,30^

### 2.3 Preparation of Na_7_Ga_13_ and NaGa_4_

Both phases were prepared from a stoichiometric mixture of the elements in sealed tantalum ampoules. Mixtures of approximately 1 g were heated in an induction coil for 1 min at 1000 °C and cooled down by removing the specimen from the coil. The samples were annealed for 7 days at 400 °C for Na_7_Ga_13_ and 350 °C for NaGa_4_ and quenched in water. The reaction products were single-phase according to PXRD and sensitive to air and moisture.

### 2.4 Characterization

#### 2.4.1 X-ray Powder Diffraction (PXRD)

All samples were characterized by the Guinier technique (Huber Image Plate Camera G670; germanium monochromator; Cu *K*_α1_ radiation; λ = 1.54056 Å; 5.0° < 2θ < 100°; step width = 0.005°). Finely ground specimens were fixed under an Ar atmosphere on the sample holder between two polyimide foils (7.5 μm, Kapton, Chemplex) using a film of vacuum grease (Lithelen, Leybold). The LaB_6_ standard NIST SRM 660a (*a* = 4.1569162(97) Å at 295.5 K) was added to the sample as a reference. The lattice parameters were refined by a least-squares method with the WinCSD program package.^31^

#### 2.4.2 Single Crystal X-ray Diffraction

A single crystal (irregular shape; *d* ≈ 0.02 mm) was fixed on top of a glass capillary (*d* = 0.1 mm) with grease and sealed in another capillary (*d* = 0.3 mm). Single-crystal X-ray diffraction data were collected using a Rigaku AFC7 diffractometer (Saturn 724+ CCD detector, Mo K_α_ radiation, λ = 0.71073 Å). An absorption correction was performed with a multi-scan procedure. The crystal structure was solved and refined using WinCSD. Details on the data collection and structure refinement are shown in Table S2; atomic coordinates and displacement parameters are listed in Table S3; selected interatomic distances and angles are listed in Tables S4–S6. The program Atoms 6.3 was used for structure drawings.^32^

#### 2.4.3 Thermal Analysis

Specimens of 30–50 mg were sealed in Nb crucibles (*d* = 5 mm; *m* = 600 mg). The closed ampoules were investigated under an Ar atmosphere with a heat-flux DTA device (Netzsch DSC 404C) from room temperature to 600 °C with heating rates between 2 and 10 °C/min.

#### 2.4.4 Scanning Electron Microscopy (SEM)

The microstructure of as-cast products was investigated with a Philips XL 30 scanning electron microscope (LaB_6_ cathode). The Na_2_Ga_7_ samples decomposed on polishing, impeding quantitative analysis by WDXS.

#### 2.4.5 Electrical Resistivity Measurements

Electrical resistivity was measured on a pressed pellet in a sapphire cell within a cryostat using a four-probe Van der Pauw method. The temperature range was *T* = 4–300 K (from −269.15 to 26.85 °C) in a zero external magnetic field. The setup was installed in an Ar-filled box.

#### 2.4.6 Magnetic Susceptibility

Magnetization was measured in a SQUID magnetometer (MPMS-XL7, Quantum Design) in the temperature range of 1.8–400 K (from −271.35 to 126.85 °C). Fields of μ_0_H from 2 mT to 7 T were applied, and the diamagnetic contribution of the sample tube was subtracted.

### 2.5 Calculation Methods

The electronic structure calculations were carried out using the all-electron full-potential local orbital FPLO method.^33^ Exchange-correlation effects were taken into account by the local density approximation to the density functional theory as parameterized by Perdew and Wang.^34^ Experimentally determined crystal structure data were used for studying the electronic structure. Na2 and Na3 atoms located on partially occupied split positions were modeled using mean positions. Additionally, atomic sites were optimized in two ways, keeping the lattice parameters constant. In one case, the Ga positions were fixed to their experimental values and only Na positions were optimized. In the second case, complete atomic-site optimization was realized. The force criterion in both cases was 10 meV Å^–1^.

## 3 Results and Discussion

### 3.1 Preparation

#### 3.1.1 Oxidation of Na_7_Ga_13_ by NH_3_

Na_2_Ga_7_ was first discovered from the reaction of Na_7_Ga_13_ and gaseous NH_3_. The reactions aimed at finding metastable compounds in the sodium–gallium system.^26−28^ When Na_7_Ga_13_ and NH_3_ were reacted in a molar ratio of 1:160 at 300 °C, the composition of the reaction product changed with the reaction time according to PXRD, allowing conclusions on the redox process. After 10 min, NaGa_4_ was the major product with detectable amounts of Na_2_Ga_7_ and remnants of the starting material (Scheme 1 and Figure S2). However, after extending the reaction time to 2 h, Na_2_Ga_7_ was the major product with only a small amount of NaGa_4_. For longer reaction times, such as 6 h, NaGa_4_ was the main product again.

In the first reaction step, Na_7_Ga_13_ is oxidized by NH_3_ to NaGa_4_, which forms together with NaNH_2_ and H_2(g)_ (eq 6). NaNH_2_ decomposes at 300 °C to elemental Na (eq 2),^35^ which reduces NaGa_4_ to Na_2_Ga_7_ (eq 3). Na_2_Ga_7_ can also be formed by comproportionation of NaGa_4_ with the remaining Na_7_Ga_13_ (eq 1). Finally, Na_2_Ga_7_ reacts with excess NH_3_ to NaGa_4_, and it is completely converted after 6 h reaction time (eq 7). Annealing experiments supported both mechanisms. When a stoichiometric mixture of finely powdered NaGa_4_ and NaNH_2_ was pressed to a pellet and annealed at 300 °C in an open tantalum crucible, Na_2_Ga_7_ was the major phase after 1 h (eqs 2 and 3). On the other hand, when a stoichiometric mixture of NaGa_4_ and Na_7_Ga_13_ was pressed to a pellet and annealed at 300 °C in a closed tantalum ampoule, Na_2_Ga_7_ formed as well as the main phase already after 1 h reaction time (eq 1).
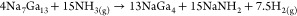
6
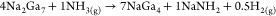
7

The oxidation of Na_2_Ga_7_ to NaGa_4_ (eq 7) is slower than the oxidation of Na_7_Ga_13_ to NaGa_4_ (eq 6) followed by the formation of Na_2_Ga_7_ either from a reaction with Na (eqs 2 and 3) or the comproportionation reaction (eq 1 and Scheme 1). Therefore, the intermediate product Na_2_Ga_7_ became the main phase after 2 h of reaction time. NaGa_4_ was the final product. Further conversion to elemental Ga was not observed after one week at 300 °C.

At room temperature, Na_7_Ga_13_ did not react with gaseous NH_3_, although the compound is highly susceptible to air and moisture. In the reaction under an NH_3_ atmosphere at 300 °C, the Na atoms are removed from the gas phase by a reaction with NH_3_ molecules to NaNH_2_ and H_2_. After raising the reaction temperature to 400 °C, the oxidation product was nano-crystalline GaN as established by broad reflections in PXRD.

#### 3.1.2 Oxidation of Na_7_Ga_13_ in Air

For PXRD measurements, specimens were enclosed in a sample holder between slightly gas-permissive Kapton foils. When a sample holder loaded with single-phase Na_7_Ga_13_ was exposed to air for 90 min, a trace amount of Na_2_Ga_7_ was detected by PXRD together with a distinct background fitting to amorphous Ga. We expected that Na_7_Ga_13_ was partially oxidized to elemental Ga followed by a comproportionation to Na_2_Ga_7_. In a control experiment, however, Na_7_Ga_13_ did not react with elemental gallium at room temperature under an Ar atmosphere. Therefore, we conclude that Na_2_Ga_7_ was directly formed from the oxidation of Na_7_Ga_13_ in air.

#### 3.1.3 Thermal Decomposition of Na_7_Ga_13_

**Figure 1:**
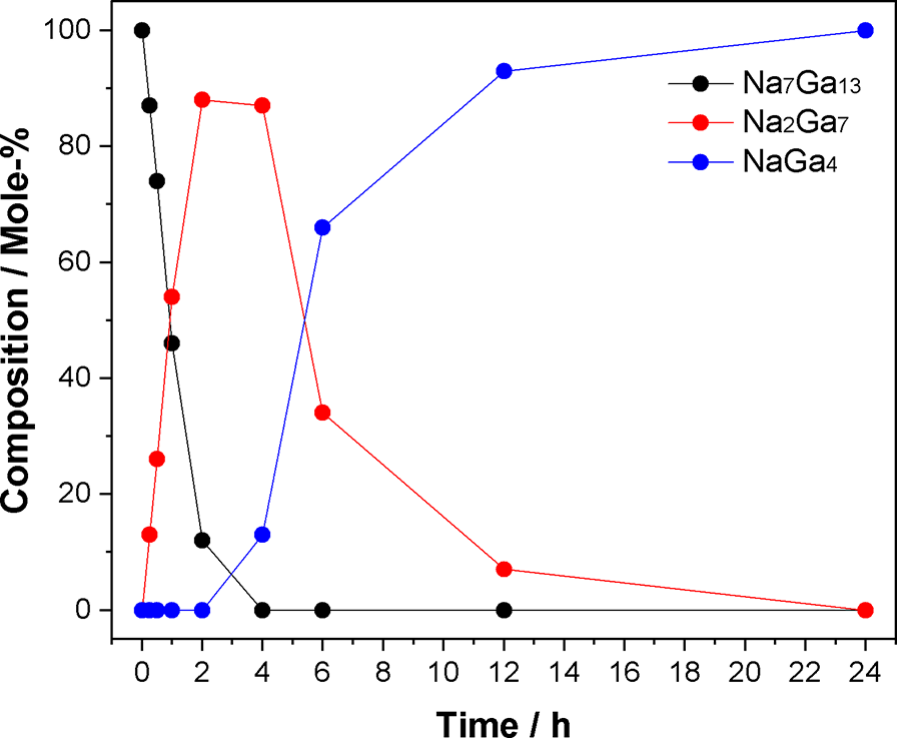
Thermal decomposition products of Na_7_Ga_13_ at 300 °C. Na_2_Ga_7_ is formed as an intermediate product and reacts further to NaGa_4_.

Thermal treatment of Na_7_Ga_13_ was performed under static vacuum at 300 °C to study the temperature stability of the phase without an oxidizing agent. Na_7_Ga_13_ has a significant sodium vapor pressure at this temperature, so thermal decomposition might have superimposed the oxidation reaction. For reaction times of up to 30 min, the starting material Na_7_Ga_13_ remained the major phase accompanied only by small amounts of Na_2_Ga_7_ and NaGa_4_. After 4 h, sodium droplets had formed in the colder part of the glass reactor. The residue consisted of Na_2_Ga_7_ as the main phase along with NaGa_4_. When the reaction time was extended to 1 day, only NaGa_4_ was detected by PXRD (Figure 1 and Table S7). The visible difference compared to the reaction in the NH_3_ atmosphere was the deposition of Na droplets at the colder part of the reactor, maintaining a constant Na vapor pressure in the system. The reaction rate is thus lower than in the NH_3_ atmosphere where the sodium partial vapor pressure is very low. The formation of Na_2_Ga_7_ from Na_7_Ga_13_ and the decomposition of Na_2_Ga_7_ to NaGa_4_ proceed in parallel (Figure 1). Consequently, single-phase Na_2_Ga_7_ was not obtained.

#### 3.1.4 Preparation of Na_2_Ga_7_ from the Elements

**Figure 2:**
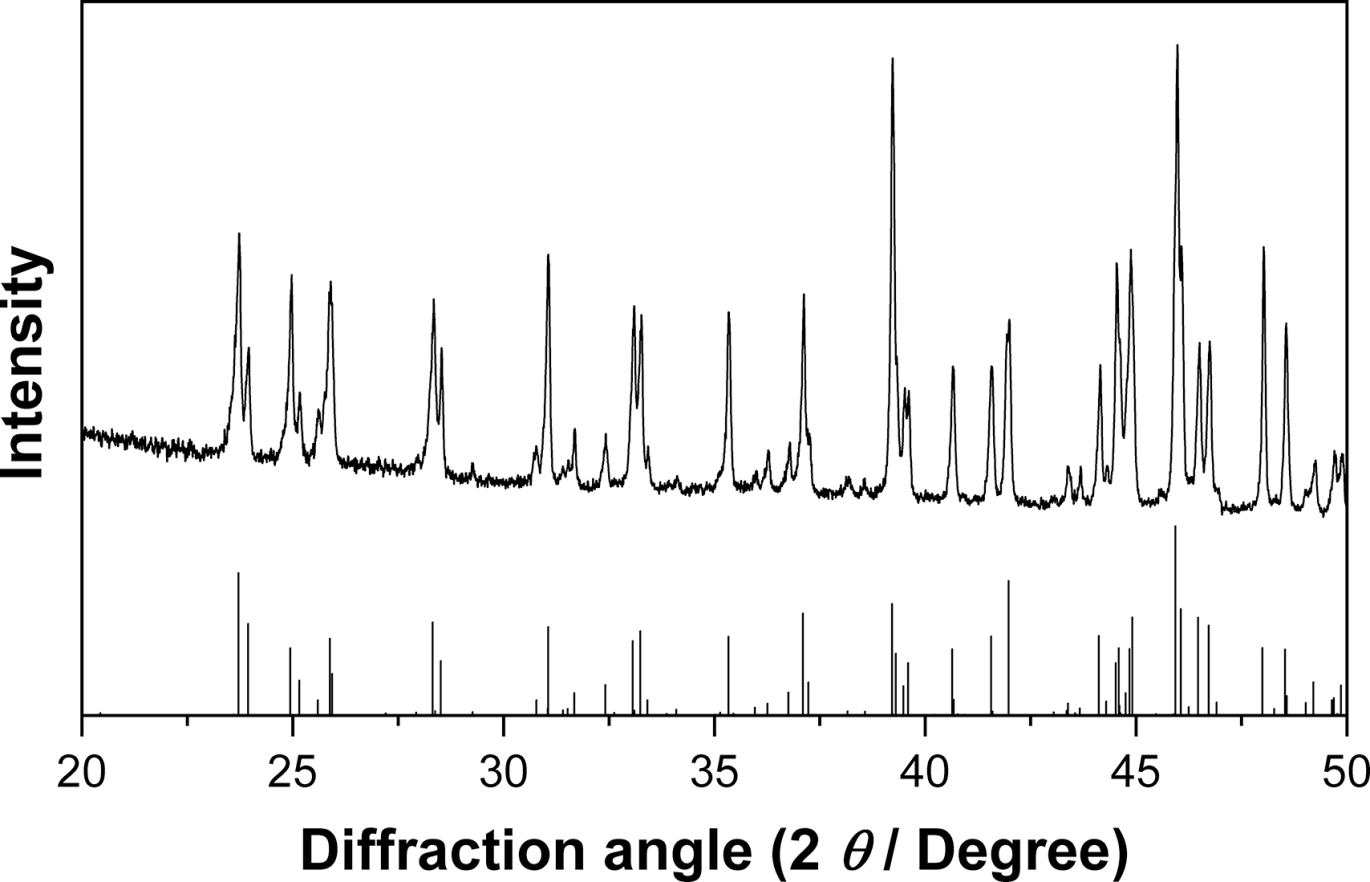
PXRD pattern (Cu *K*_α1_, λ = 1.540598 Å) of Na_2_Ga_7_ after preparation from the elements and annealing at 300 °C for 7 days. The calculated reflection intensities (bottom) are based on the structure model obtained from single-crystal diffraction data.

When a stoichiometric mixture of Na_2_Ga_7_ was melted and cooled down to room temperature within 2 min, the product consisted of Na_7_Ga_13_, NaGa_4_, and Ga. Consequently, Na_2_Ga_7_ forms incongruently. Annealing for 7 days at temperatures between 200 and 450 °C always resulted in a single-phase product Na_2_Ga_7_ (Figure 2) with the same lattice parameters (Table S8a). A possible homogeneity range of Na_2_Ga_7_ was investigated by annealing samples in the two-phase regions NaGa_4_/Na_2_Ga_7_ and Na_2_Ga_7_/Na_7_Ga_13_ at 200 or 300 °C. In all cases, the lattice parameters of Na_2_Ga_7_ were constant within an experimental error (Table S8b). Moreover, the lattice parameters of Na_2_Ga_7_ did not change for the different preparation methods, such as the direct synthesis, solid-state reaction, thermal decomposition, or redox preparation (Table S8c). From these results, Na_2_Ga_7_ is an equilibrium phase with a constant composition.

### 3.2 Phase Diagram

**Figure 3:**
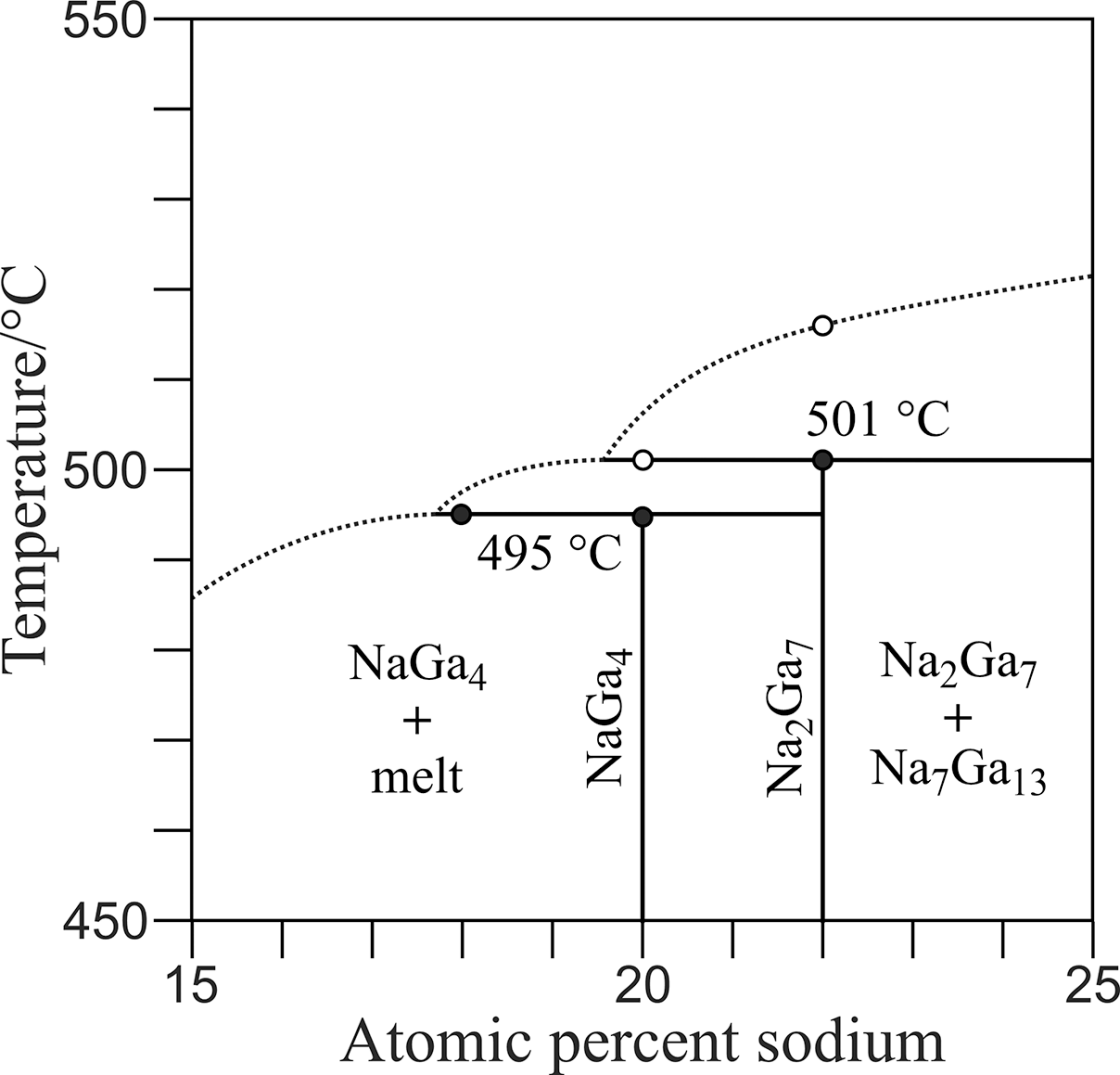
Phase relations of Na_2_Ga_7_ in the Na–Ga system. The slope of the liquidus curve is estimated. Filled circles represent onset temperatures, and empty circles show peak temperatures in DTA experiments.

In the Na–Ga system, the binary phases NaGa_4_ (20 at-% Na), Na_7_Ga_13_ (35 at-% Na), and Na_22_Ga_39_ (36 at. % Na) have been reported. The latest report on the phase diagram only considered the phases NaGa_4_ and Na_22_Ga_39_.^36,37^ NaGa_4_ should form peritectically at 504 °C from Na_22_Ga_39_ and the melt. In this work, the phase relations of Na_2_Ga_7_ (22 at-% Na) were investigated in the region of 20–35 at. % Na from DTA and PXRD data (Figure 3). A DTA measurement of single-phase NaGa_4_ revealed a strong endothermic effect at 495 °C, which is attributed to the peritectic reaction NaGa_4_ → Na_2_Ga_7_ + L. Na_2_Ga_7_ also decomposes peritectically: the endothermic effect at 501 °C corresponds to the reaction Na_2_Ga_7_ → Na_7_Ga_13_ + L. Due to the slight difference in decomposition temperatures, only a small fraction of the liquidus curve is associated with the formation of Na_2_Ga_7_. Therefore, the phase was not detected in the non-equilibrium mixtures obtained by fast cooling from the melt. The peritectic isotherm of Na_2_Ga_7_ was observed by thermal effects between 21 and 35 at. % Na. The decomposition temperature of Na_7_Ga_13_ was found to be 549 °C.

### 3.3 Crystal Structure

#### 3.3.1 Structure Determination

**Figure 4:**
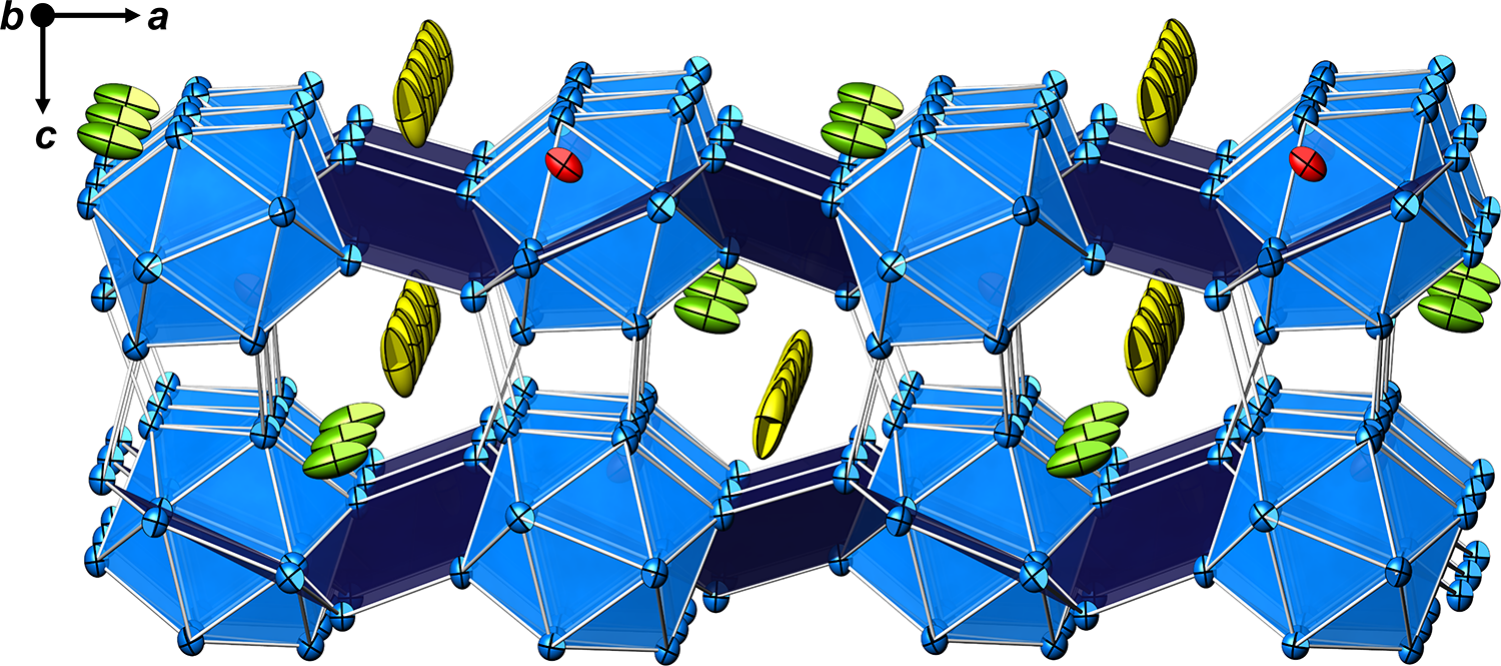
Ordered crystal structure model of Na_2_Ga_7_ along [010]. Without split atoms, the ADPs of Na2 (green) and Na3 (yellow) reveal large and elongated ellipsoids. Na1 (red) occupies caves between adjacent icosahedra within the layer.

The first preliminary structure model of Na_2_Ga_7_ was established from PXRD data in the space group *Cmce*. While the Ga positions were well defined, the Na positions were found to be highly disordered. Single-crystal X-ray diffraction experiments revealed reflections violating the *C*-centered lattice. Analysis of extinction conditions yielded the possible space groups *Pnma* and *Pn*2_1_*a*. The structure solution in *Pnma* directly revealed an icosahedral cluster unit (Ga1–Ga8), which is connected to a framework via intercluster bonds and by four-bonded atoms (Ga9 and Ga10). The difference Fourier map indicated a series of additional electron density peaks ∼3 Å away from the Ga atoms. The three strongest ones were assigned to Na1–Na3 with full occupancy, so the overall composition was Na_2_Ga_7_ (*Z* = 8). The structure refinement with an anisotropic approximation of the atomic displacement parameters (ADPs) yielded a residual of *R*_F_ = 0.07. For all Ga atoms, the refined ADPs were nearly isotropic, and the *U*_eq_ values were similar for different positions. For the Na atoms, the ADPs of Na1 were isotropic as well, but the ADPs of Na2 and Na3 yielded large and elongated ellipsoids (Figure 4). By introducing split positions Na2*a*/Na2*b* and Na3*a*/Na3*b* with isotropic ADPs, the residual value dropped to *R*_F_ = 0.054. For both split positions, the sum of occupancies was 1 within one standard deviation. In the final refinement cycle, the restraints *occ*(Na2*a*) + *occ*(Na2*b*) = 1 and *occ*(Na3*a*) + *occ*(Na3*b*) = 1 were applied. The so-obtained composition of the unit cell, that is, Na_16_Ga_56_, is electronically balanced according to [Na^+^]_16_[(Ga_12_)^2–^]_4_[Ga^–^]_8_, when the icosahedra are considered as [(12b)Ga_12_]^2–^*closo*-clusters^38,39^ and the four-bonded atoms as [(4b)Ga]^−^ anions.^40−42^ The experimental data with mean positions for Na2 and Na3 were used as a model for structure optimization using the FPLO code (Table S9). In the optimized structure model, the Ga and Na1 positions were nearly identical to the experimental ones, while the calculated positions for Na2 and Na3 center the respective split positions from the experiment.

#### 3.3.2 Crystal Structure Description

**Figure 5:**
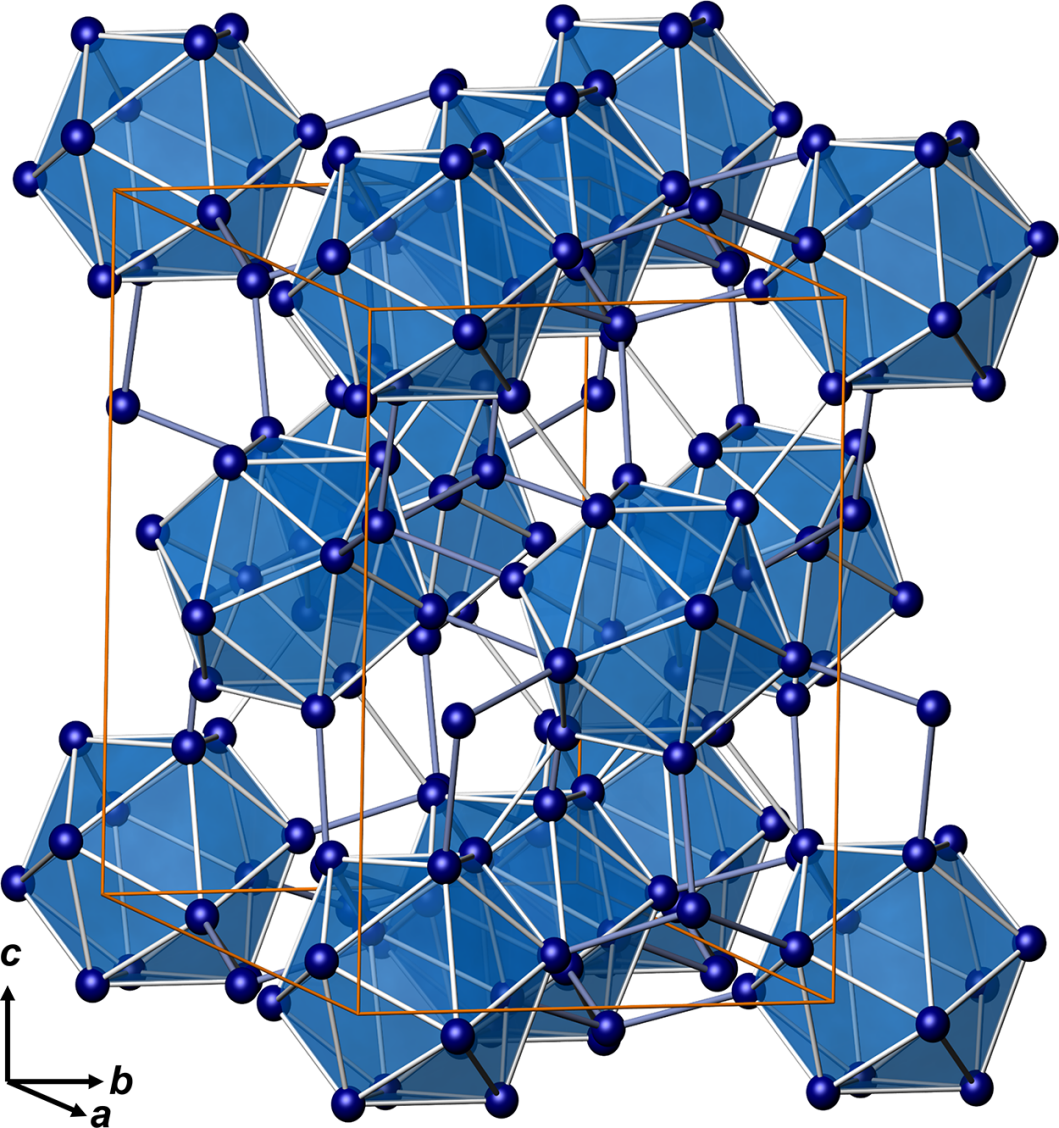
Ga framework in Na_2_Ga_7_ with interconnected icosahedral units and four-bonded atoms. The icosahedra form a distorted face-centered arrangement.

**Figure 6:**
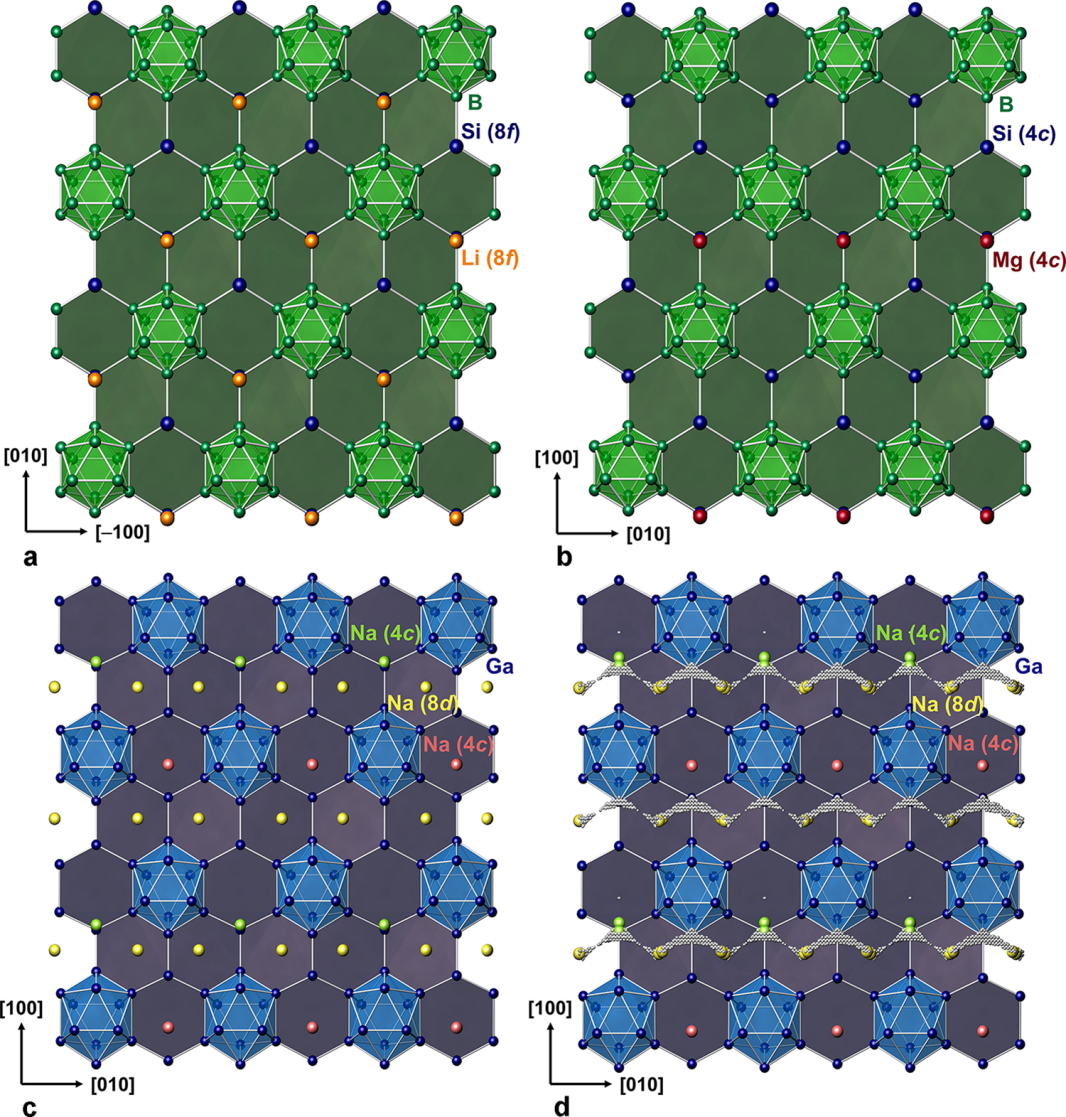
Structure motif of puckered hexagon layers with icosahedra embedded in the compounds with an isostructural framework: (a) Li_2_B_12_Si_2_; (b) MgB_12_Si_2_; (c) Na_2_Ga_7_, optimized positions; and (d) Na_2_Ga_7_, positions from structure refinement. Small white spheres represent regions with *d* ≥ 3.0 Å to the Ga atoms.

**Figure 7:**
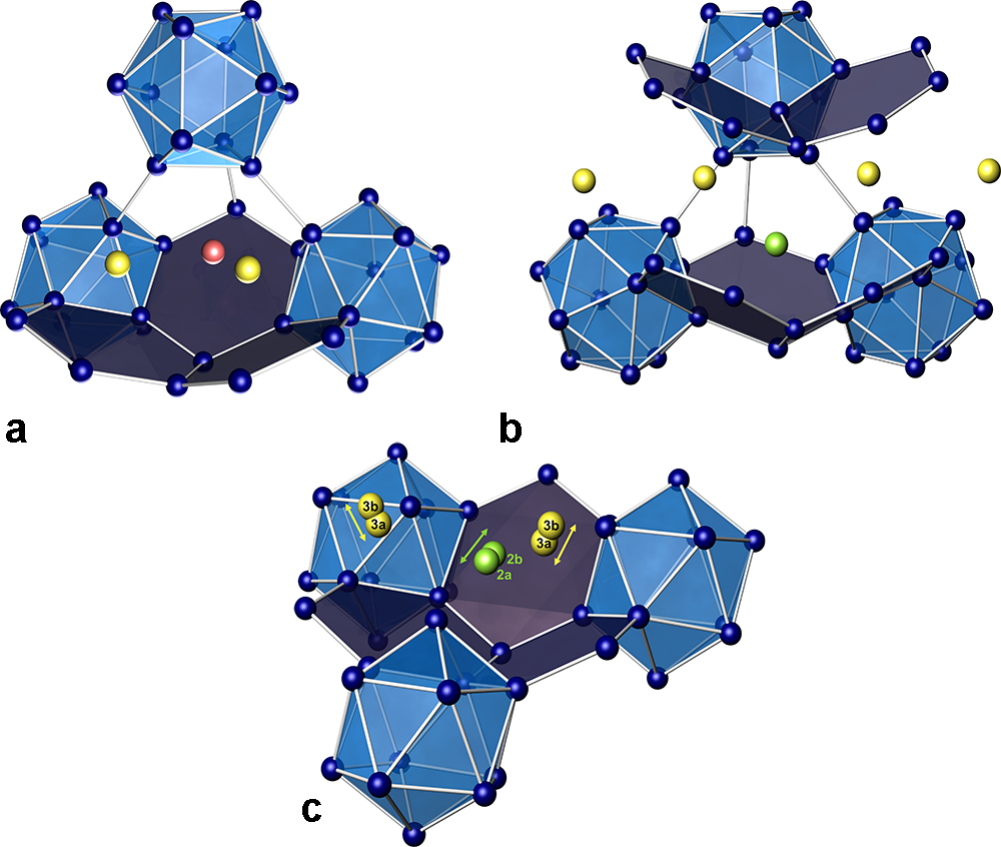
Local atomic arrangement in Na_2_Ga_7_: (a) Na1 (red) and Na3 (yellow); (b) Na2 (green) and Na3 (yellow); and (c) split positions of Na2 (green) and Na3 (yellow) caused by mutual repulsion.

The crystal structure of Na_2_Ga_7_ (*Z* = 8) is closely related to that of the borosilicides MgB_12_Si_2_^43^ and Li_2_B_12_Si_2_^44^ (*Z* = 4). The compounds feature topologically the same anionic framework of interconnected icosahedral clusters and four-bonded atoms (Figure 5). The [(12b)B_12_]^2–^ Wade clusters in the borosilicides correspond to [(12b)Ga_12_]^2–^ icosahedra in Na_2_Ga_7_ and the [(4b)Si]^0^ to the [(4b)Ga]^−^ units. The framework can be described by strongly puckered hexagon layers perpendicular to [001] containing the icosahedra interlinked by their six-membered rings in a chair conformation (Figure 6). The hexagon layers show an AA stacking in which the icosahedron positions of adjacent layers shift by 0.5 *b*. Within each layer, always, three adjacent hexagons form troughs, which is convenient to accommodate the cations. In Li_2_B_12_Si_2_ (space group *Cmce*), the troughs are filled by Li atoms at Wyckoff position 8*f* (Figure 6a). As MgB_12_Si_2_ comprises only half the cations, only half of the troughs are filled, and the symmetry decreases. The central trough position 8*f* in *Cmce* splits into two 4*c* positions in *Pnma*. In MgB_12_Si_2_, one of the 4*c* positions is filled by Mg atoms, while the other remains empty (Figure 6b). Na_2_Ga_7_, on the other hand, contains twice as many cations as Li_2_B_12_Si_2_, and the symmetry is *Pnma*. Na1 and Na2 fill both 4*c* positions, but they are shifted at different distances from the central trough position (Figure 6c). The different positions of Na1 and Na2 result from the space requirement of Na3 at site 8*d*. The Na3 atoms form linear chains in the channels along [010] and have no counterpart in the borosilicides. Their position could be equally described by position 8*e* in *Cmce*. Therefore, the *Cmce* symmetry is only broken by the different environments of Na1 and Na2. The Na1 atoms are shifted into a narrow cave between three icosahedra, confined by one six-membered ring and two five-membered rings (Figure 7a). At this position, the Na1 atom has a short distance of *d* = 1.37 Å to the center of the six-membered ring. The adjacent cave on the opposite side of the six-membered ring remains empty because the steric hindrance by Na1 prevents the accommodation of another Na atom. Therefore, the Na2 atoms shifted less from the central trough position (Figure 7b), leading to a repulsive interaction with the Na3 atoms. The split positions of Na2 and Na3 compromise their mutual space demand. When the Na2 atom occupies the position Na2a close to the central trough, the Na3 atom shifts upward to Na3b (Figure 7c). Vice versa, when the Na3 atom occupies position Na3a, the Na2 atom shifts to Na2b toward the narrow cave. The positions Na2a and Na3a have a short distance of 3.1 Å, but, with a shift either to Na2b or Na3b, Na2 and Na3 achieve a distance of 3.5 Å. The free volume in the framework available for Na2 and Na3 atoms can be visualized by space increments with a distance of *d* ≥ 3.0 Å to the closest Ga atom (Figure 6d, white spheres). The refined split positions of Na2 and Na3 cover areas allowing a maximum distance between adjacent Na atoms.

**Figure 8:**
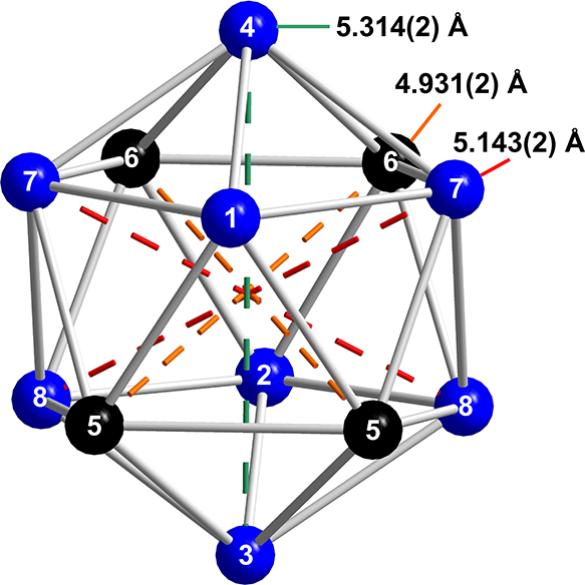
Distorted Ga icosahedron in Na_2_Ga_7_ with different distances of atoms in *trans*-positions. Atoms connected with four-bonded Ga atoms are drawn in blue with atoms forming exohedral bonds to neighboring icosahedra in black.

Different to the [B_12_]^2–^ clusters in the borosilicides Li_2_B_12_Si_2_ and MgB_12_Si_2_, the [Ga_12_]^2–^ icosahedra in Na_2_Ga_7_ are distorted (Table S4 and Figure S3). The endohedral interatomic distances range from 2.611(2) to 2.882(2) Å and the distances between *trans*-position atoms from *d*(Ga5–Ga6) = 4.931(2) Å to *d*(Ga3–Ga4) = 5.314(2) Å (Figure 8). In addition, the four-bonded atoms in all compounds deviate from a tetrahedral coordination. In the case of Na_2_Ga_7_, the bond angles vary from 98.95(3) to 130.36(9)° (Table S6). As expected, the exohedral two-center Ga–Ga bonds (*d̅*_exo_ = 2.57 Å) in Na_2_Ga_7_ are shorter than the endohedral multicenter bonds (*d̅*_endo_ = 2.69 Å). The average interatomic distance in the Ga framework of Na_2_Ga_7_ (*d̅* = 2.66 Å) is larger than in NaGa_4_ (*d̅* = 2.61 Å) but smaller than in Na_7_Ga_13_ (*d̅* = 2.71 Å), which is in accordance with the average formal charge per Ga atom.

### 3.4 Electronic Structure

**Figure 9:**
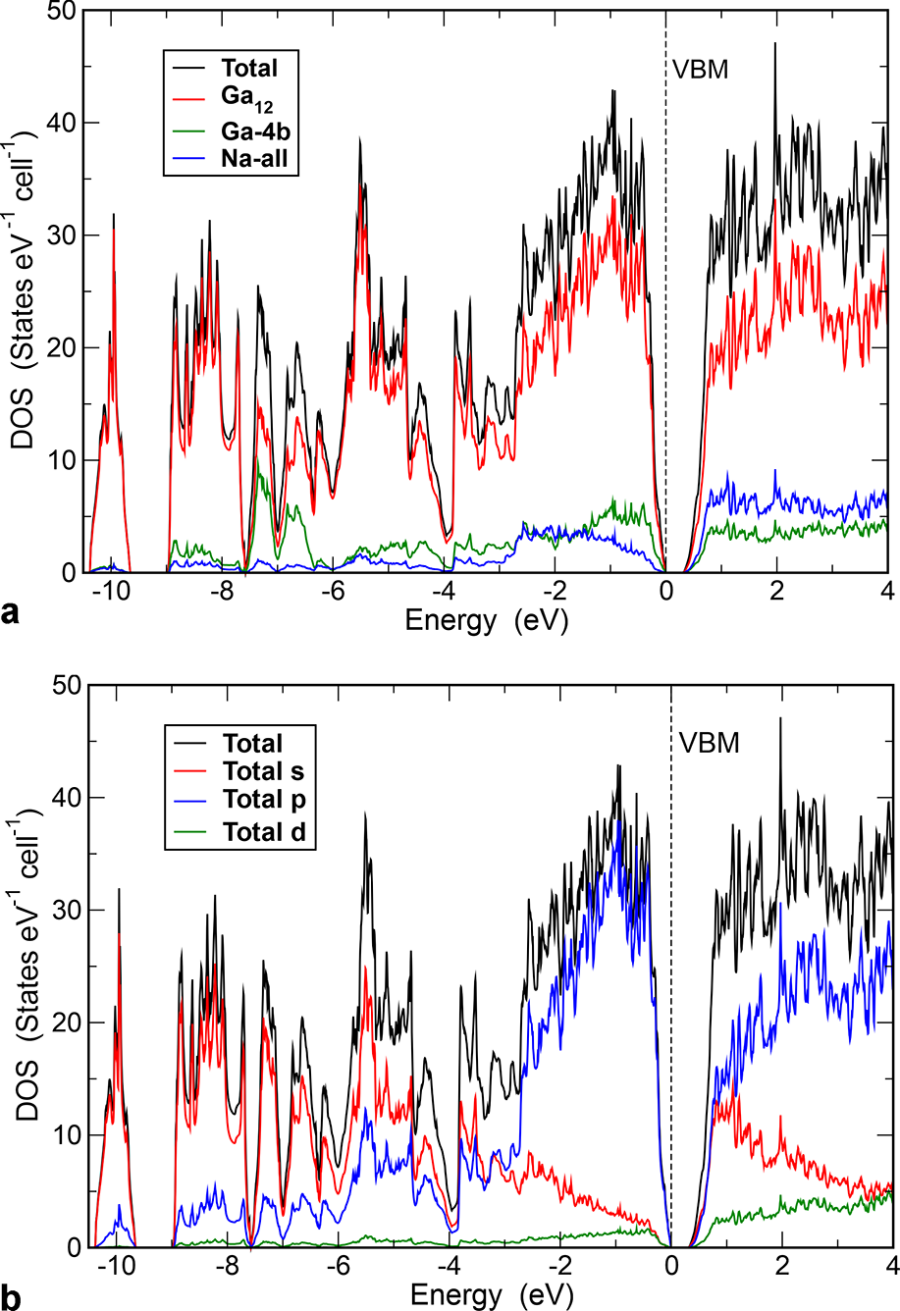
Computed electronic density of states (DOS) for Na_2_Ga_7_: (a) total and angular momentum-resolved DOS; (b) total and structural unit-projected DOS.

The computed electronic density of states (DOS) of Na_2_Ga_7_ reveals a band gap of 0.29 eV (Figure 9), which is in accordance with the Zintl and Wade counting rules predicting an electron-balanced composition for (12b)Ga_12_ icosahedral *closo*-clusters and (4b)Ga atoms:
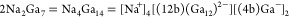


In the total DOS, the *s* states dominate the energy range between −10.5 and −3.5 eV, while the *p* states dominate the energy range above. As expected, *d*-state contributions in the range from −9 to 0 eV are small (Figure 9a). The Ga_12_ icosahedron is obviously the dominant substructure of the DOS (Figure 9b). The states between −10.5 and −7.5 eV indicate mainly intra- and inter-icosahedron *s*–*s* bonding contributions. The four-bonded [Ga]^−^ species have their largest contributions between −7.5 and −6.5 eV. The contributions of the Na atoms to the total DOS are small and distributed over the whole energy range. This is consistent with Na atoms transferring electrons to the Ga framework.

The structurally related ternary borosilicides also have electron-balanced compositions:^43,44^
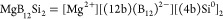

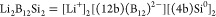


However, this counting scheme does not strictly apply to all alkali-metal gallides. For gallides of lithium, both electron-deficient and electron-excess compositions have been reported as well (p^+^ stands for a positive and e^–^ for a negative charge).^13,14,45^ Such cases are worth investigating in depth.
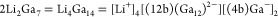

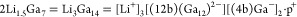

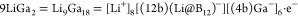


In the related gallides of the heavier alkali metals, the Ga_12_ icosahedra form multi-centered bonds:^15,16,46^
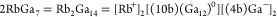

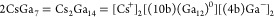


### 3.5 Physical Properties

The electrical resistivity ρ(*T*) of the specimens shows linear temperature dependence with a positive temperature coefficient, which is characteristic of metallic-type compounds (Figure S4a). This result contrasts the band structure calculation and is not expected from the electron-balanced composition. Due to the high sensitivity of the compound toward air and moisture, the metallic behavior may be caused by elemental Ga, which forms on the surface during the oxidation or hydrolysis of the sample in air. PXRD measurements of the specimen after the conductivity measurement did not reveal any other foreign phase formed during the measurement. The relatively high resistivity values would be consistent for a sintered pressed pellet. The small drop of resistivity below *T* ≈ 8 K (−265.15 °C) can be attributed to the presence of Ga, which is superconducting at these temperatures.^47^ No sign of a phase transition was observed in the electrical resistivity in the measured temperature range, which might be considered as a contradiction for the presence of Ga forming low-temperature allotropes. A test measurement on elemental Ga (Figure S4b) did not reveal thermodynamic transitions either, probably for kinetic reasons.

The magnetization *M*(*T*) of the sample was measured in the temperature range from *T* = 1.8 to 400 K (−271.35 °C to 126.85 °C) in magnetic fields μ_0_*H* = 0.002, 0.1, 3.5, and 7 T. The magnetic moment of the sample holder was subtracted from the measured data, and the magnetic susceptibility χ = *M*/*H* was calculated. The magnetic susceptibility χ(*T*) is temperature-independent and diamagnetic (Figure S5) with the value χ = −1.55 × 10^–4^ emu/mol per formula unit. The experimental value agrees within 1% with the calculated value from diamagnetic increments for Na^+^ and elemental Ga.^48^ The absence of Pauli paramagnetism supports the assumption that Na_2_Ga_7_ is non-metallic. The observed superconductivity below *T* ≈ 8 K (−265.15 °C) is attributed to the presence of elemental Ga on the surface since the Meissner fraction of the signal is low (∼0.015%). According to the reported data, the allotropes β-, γ-, and δ-Ga show superconducting transitions at 5.9–6.2 K (from −267.25 to −266.95 °C), 6.9–7.6 K (from −266.25 to – 265.55 °C), and 7.85 K (−265.3 °C), respectively.^47^

## 4 Conclusions

Na_2_Ga_7_ is a new equilibrium line phase in the Na–Ga system, forming peritectically from Na_7_Ga_13_ and the melt. The compound is highly air and moisture-sensitive and has been prepared either by annealing the elemental components or by redox reactions. With its framework of icosahedral Ga_12_ clusters and four-bonded Ga atoms, Na_2_Ga_7_ extends the series of the structurally related binary gallides LiGa_2_, Li_3_Ga_14_, RbGa_7_, and CsGa_7_. Moreover, Na_2_Ga_7_ constitutes a filled variant of the Li_2_B_12_Si_2_ type of structure, while the higher content of cations leads to a complex structural organization. Na_2_Ga_7_ is electronically balanced according to the formula [Na^+^]_4_[(Ga_12_)^2–^][Ga^–^]_2_, diamagnetic, and, from electronic structure calculations, a semiconductor with a band gap of 0.29 eV.

## Supporting Information Available

Tables with crystallographic data from diffraction experiments and optimization, experimental results from SEM and thermal decomposition, resistivity and susceptibility measurements, and structural relation between α-tetragonal boron and Na_2_Ga_7_ (PDF)
