# Supplementary material for: Na2Ga7: A Zintl–Wade Phase Related to “α-Tetragonal Boron”
Source: Inorg Chem. 2023 May 25;62(23):9054–62. doi: 10.1021/acs.inorgchem.3c00790 (PMC10265698; doi:10.1021/acs.inorgchem.3c00790)
Supplement: Supplementary file 1 — ic3c00790_si_001.pdf [file ic3c00790_si_001.pdf]

Supporting Information

Na_2Ga_7: A Zintl-Wade phase related to "α-tetragonal boron"

Chia-Chi Yu[1], Alim Ormeci[1], Igor Veremchuk[2], Xianjuan Feng[1], Yurii Prots[1], Mitja Krnel[1], Primož Koželj[3], Marcus Schmidt[1], Ulrich Burkhardt[1], Bodo Böhme[1], Lev Akselrud[1,4], Michael Baitinger[1], Yuri Grin[1]*

[1]Max-Planck-Institut für Chemische Physik fester Stoffe, Nöthnitzer Str. 40, 01187 Dresden, Germany

[2]Helmholtz-Zentrum, Dresden-Rossendorf, Bautzener Landstraße 400, 01328 Dresden, Germany

[3]Jozef Stefan Institute, P.O. Box 3000, 1001 Ljubljana, Slovenia

[4]Ivan Franko Lviv National University, Kyryla i Mefodia St. 57, 29005 Lviv, Ukraine

*grin@cpfs.mpg.de

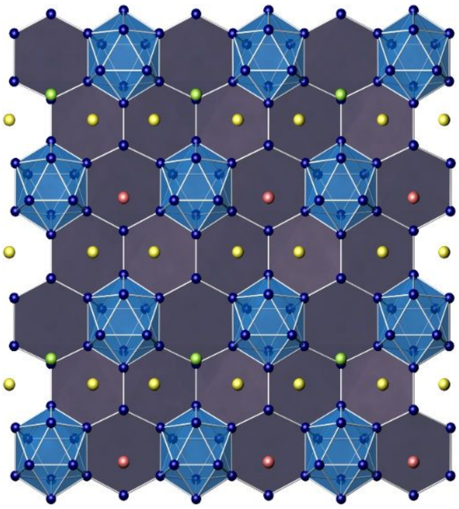

Table of content:

SEM of Na_2Ga_7 formed by oxidation	S1
XRPD of samples after reaction of Na_7Ga_{13} in $NH_{3(g)}$	S2
Lattice parameters of β-$NaGaO_2$ and β-Ga_2O_3	S3
Crystallographic data for Na_2Ga_7	S4
Atomic coordinates and displacement parameters	S5
Selected interatomic distances	S6
Selected bond angles	S8
Thermal decomposition products of Na_7Ga_{13}	S9
Lattice parameters of Na_2Ga_7	S10
Optimized crystal structure of Na_2Ga_7	S11
Schlegel diagram of the Ga_{12} icosahedron of Na_2Ga_7	S12
Resistivity measurement of Na_2Ga_7	S13
Susceptibility measurement of Na_2Ga_7	S14
References	S15

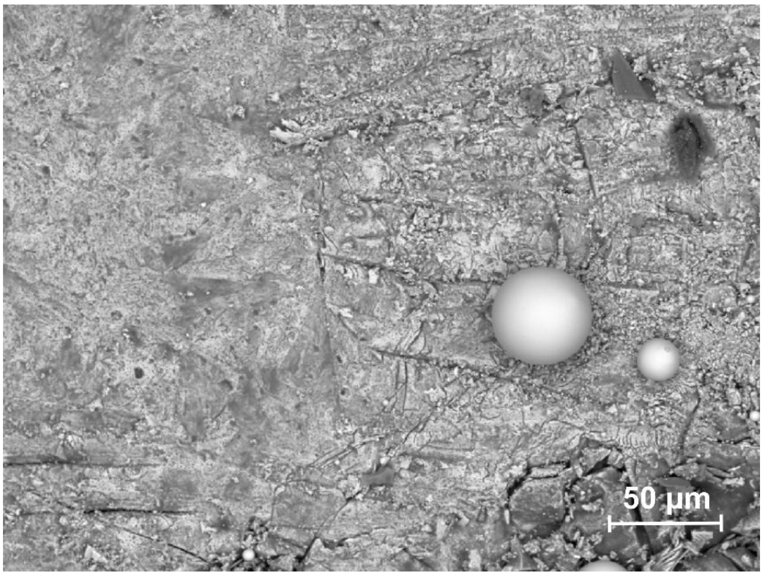

Figure S1. SEM image of the product from the reaction of Na_7Ga_{13} with $NH_{3(g)}$; BSE contrast. Droplets of elemental Ga segregated during the oxidation process.

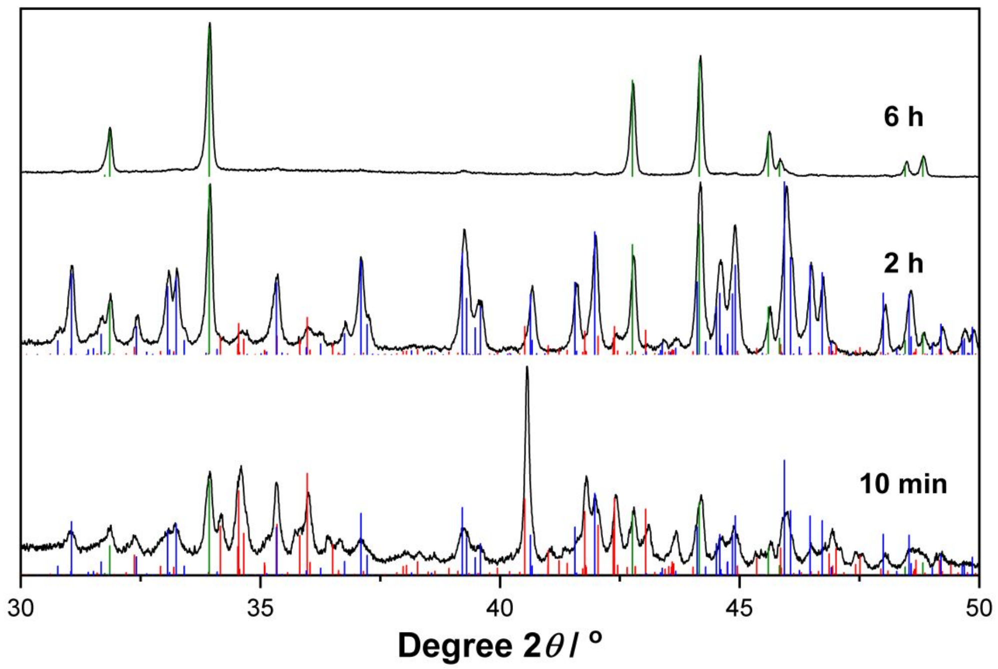

Figure S2. XRPD patterns of samples after reacting Na_7Ga_{13} in $NH_{3(g)}$ atmosphere for different times at 300 °C. The calculated reflection intensities for Na_7Ga_{13} (red), Na_2Ga_7 (blue), and $NaGa_4$ (green) are based on the structure models obtained from the SCXRD data.

Table S1. lattice Refined parameters of β-$NaGaO_2$ and β-Ga_2O_3. For β-$NaGaO_2$ the same set of reflections was taken for the products prepared at different temperatures.

**Phase**	**β-$NaGaO_2$**			**β-Ga_2O_3**	
***T* / °C**	300	800	Ref. 1	800	Ref. 2
***a* / Å**	5.516(2)	5.508(4)	5.498(1)	12.2332(5)	12.2259(4)
***b* / Å**	7.210(2)	7.215(3)	7.206(1)	3.0403(2)	3.04118(9)
***c* / Å**	5.3017(9)	5.299(2)	5.298(1)	5.8087(2)	5.8094(2)
**β / °**	–	–	–	103.845(4)	103.846(2)
***V* / Å³**	210.8(1)	210.6(2)	209.90(6)	209.76(3)	209.72(2)

Table S2. Crystallographic data for Na_2Ga_7.

Composition	Na_2Ga_7
Molar mass / g mol^{-1}	534.039
Crystal system; space group	Orthorhombic, *Pnma* (no. 62)
a / Å	14.8555(8)
b / Å	8.6814(4)
c / Å	11.6084(9)
V / Å^3	1497.0(3)
Z; ρ_{calc} / (g cm^{-3})	8; 4.739
Diffractometer	Rigaku AFC7, Saturn 724+CCD detector
λ / Å	0.71073 (Mo$K\alpha$)
Temperature / K	295
Crystal shape	irregular
Crystal size / mm	0.070 × 0.055 × 0.050
μ / mm^{-1}	26.16
Absorption correction	multi-scan
2θ range / deg.	1.4–33.6
Indexes ranges	$-18 \le h \le 22$, $-12 \le k \le 13$, $-18 \le l \le 10$
F(000) / e	1912
Reflection measured, independent	12795; 2952 [R_{int} = 0.05]
Reflections used for refinement	2149
Parameters refined	93
Goof	1.030
R_1, $F_o > 2\sigma F_o$	0.0542
R_1, all data	0.0873

Table S3. Atomic coordinates and displacement parameters (in Å^2) for Na_2Ga_7.

**Atom**	**Site**	**Occup.**	***x***	***y***	***z***	U_{eq}	U_{11}	U_{22}	U_{33}	U_{12}	U_{13}	U_{23}
Ga1	4*c*	1	0.3496(1)	1/4	0.1742(2)	0.0122(4)	0.0121(7)	0.0089(7)	0.0155(8)	0	−0.0015(6)	0
Ga2	4*c*	1	0.1358(1)	1/4	0.8183(2)	0.0147(5)	0.0145(7)	0.0145(8)	0.0151(8)	0	−0.0005(6)	0
Ga3	4*c*	1	0.0728(1)	1/4	0.0485(2)	0.0145(5)	0.0103(7)	0.0151(8)	0.0180(8)	0	0.0008(6)	0
Ga4	4*c*	1	0.4231(1)	1/4	0.9556(2)	0.0137(5)	0.0117(7)	0.0117(7)	0.0177(8)	0	0.0013(6)	0
Ga5	8*d*	1	0.19608(8)	0.0888(2)	0.1627(1)	0.0130(3)	0.0147(5)	0.0109(5)	0.0133(5)	0.0009(4)	0.0003(4)	0.0001(4)
Ga6	8*d*	1	0.20495(8)	0.5990(2)	0.3314(1)	0.0136(3)	0.0137(5)	0.0114(5)	0.0156(5)	−0.0001(4)	0.0010(4)	0.0009(4)
Ga7	8*d*	1	0.33576(8)	0.0042(2)	0.0417(1)	0.0128(3)	0.0140(5)	0.0089(5)	0.0155(5)	−0.0008(4)	−0.0003(4)	0.0007(4)
Ga8	8*d*	1	0.34511(9)	0.5053(2)	0.4518(1)	0.0146(3)	0.0162(5)	0.0107(5)	0.0169(6)	0.0010(4)	0.0010(4)	0.0004(4)
Ga9	4*c*	1	0.4128(1)	1/4	0.3810(2)	0.0132(5)	0.0124(7)	0.0126(8)	0.0144(7)	0	0.0002(6)	0
Ga10	4*c*	1	0.0902(1)	1/4	0.6032(2)	0.0138(5)	0.0137(7)	0.0101(7)	0.0175(8)	0	−0.0001(6)	0
Na1	4*c*	1	0.2124(5)	1/4	0.3908(7)	0.020(2)	0.028(4)	0.010(3)	0.020(3)	0	−0.008(3)	0
Na2a	4*c*	2/3	0.411(1)	1/4	0.635(1)	0.025(3)	–	–	–	–	–	–
Na2b	4*c*	1/3	0.380(3)	1/4	0.626(3)	0.034(7)	–	–	–	–	–	–
Na3a	8*d*	1/2	0.0047(8)	0.023(2)	0.283(1)	0.020(3)	–	–	–	–	–	–
Na3b	8*d*	1/2	0.0125(8)	0.046(2)	0.318(1)	0.022(3)	–	–	–	–	–	–

U_{eq} is defined as one third of the trace of the orthogonalized U_{ij} tensor, which is exp $(-2\pi^2 [h^2a^{*2}U_{11} + ... + 2\,h\,k\,a^*\,b^*\,U_{12}])$

Table S4. Ga–Ga interatomic distances in one unit-cell of Na_2Ga_7.

**Ga–Ga**	***d* / Å**		**Ga–Ga**	***d* / Å**	
Ga1 – Ga4	2.763(3)	×2	Ga5 – Ga5	2.800(3)	×4
– Ga5	2.679(2)	×8	– Ga6	2.552(2)	×4
– Ga7	2.639(2)	×8	– Ga7	2.611(2)	×8
– Ga9	2.578(3)	×4	– Ga8	2.626(2)	×4
Ga2 – Ga3	2.832(3)	×2	Ga6 – Ga6	2.623(3)	×4
– Ga6	2.709(2)	×8	– Ga7	2.647(2)	×4
– Ga8	2.646(2)	×8	– Ga8	2.636(2)	×8
– Ga10	2.588(2)	×4	Ga7 – Ga8	2.882(2)	×6
Ga3 – Ga5	2.659(2)	×8	– Ga10	2.568(2)	×8
– Ga8	2.695(2)	×4	Ga8 – Ga9	2.570(2)	×8
– Ga9	2.514(2)	×2			
Ga4 – Ga6	2.723(2)	×8			
– Ga7	2.691(2)	×4			
– Ga10	2.574(2)	×2			

Table S5. Ga–Ga–Ga bond angles in the icosahedron of Na_2Ga_7.

∡Ga–Ga–Ga	deg / °		∡Ga–Ga–Ga	deg / °	
Ga4–**Ga1**–Ga5	106.89(3)	×2	Ga2–**Ga6**–Ga4	113.99(4)	×2
Ga4–**Ga1**–Ga7	59.69(3)	×2	Ga2–**Ga6**–Ga6	61.05(4)	×2
Ga5–**Ga1**–Ga5	63.01(4)	×1	Ga2–**Ga6**–Ga7	113.69(5)	×2
Ga5–**Ga1**–Ga7	58.80(4)	×2	Ga2–**Ga6**–Ga8	59.31(4)	×2
Ga5–**Ga1**–Ga7	109.10(4)	×2	Ga4–**Ga6**–Ga6	61.22(4)	×2
Ga7–**Ga1**–Ga7	107.95(5)	×1	Ga4–**Ga6**–Ga7	60.11(4)	×2
Ga3–**Ga2**–Ga6	103.62(3)	×2	Ga4–**Ga6**–Ga8	114.79(6)	×2
Ga3–**Ga2**–Ga6	58.83(3)	×2	Ga6–**Ga6**–Ga7	108.12(7)	×2
Ga6–**Ga2**–Ga6	57.91(4)	×1	Ga6–**Ga6**–Ga8	107.98(7)	×2
Ga6–**Ga2**–Ga8	58.98(4)	×2	Ga7–**Ga6**–Ga8	66.12(5)	×2
Ga6–**Ga2**–Ga8	105.21(5)	×2	Ga1–**Ga7**–Ga4	62.44(3)	×2
Ga8–**Ga2**–Ga8	106.88(5)	×1	Ga1–**Ga7**–Ga5	61.36(4)	×2
Ga2–**Ga3**–Ga5	104.07(3)	×2	Ga1–**Ga7**–Ga6	107.70(5)	×2
Ga2–**Ga3**–Ga8	57.13(3)	×2	Ga1–**Ga7**–Ga8	106.32(5)	×2
Ga5–**Ga3**–Ga5	63.53(4)	×1	Ga4–**Ga7**–Ga5	111.09(6)	×2
Ga5–**Ga3**–Ga8	58.74(4)	×2	Ga4–**Ga7**–Ga6	61.35(4)	×2
Ga5–**Ga3**–Ga8	108.14(4)	×2	Ga4–**Ga7**–Ga8	108.19(5)	×2
Ga8–**Ga3**–Ga8	104.09(5)	×1	Ga5–**Ga7**–Ga6	103.14(5)	×2
Ga1–**Ga4**–Ga6	102.15(3)	×2	Ga5–**Ga7**–Ga8	56.86(4)	×2
Ga1–**Ga4**–Ga7	57.87(3)	×2	Ga6–**Ga7**–Ga8	56.76(4)	×2
Ga6–**Ga4**–Ga6	57.57(4)	×1	Ga2–**Ga8**–Ga3	64.04(3)	×2
Ga6–**Ga4**–Ga7	58.53(4)	×2	Ga2–**Ga8**–Ga5	110.46(7)	×2
Ga6–**Ga4**–Ga7	104.01(4)	×2	Ga2–**Ga8**–Ga6	61.71(4)	×2
Ga7–**Ga4**–Ga7	105.00(5)	×1	Ga2–**Ga8**–Ga7	108.35(5)	×2
Ga1–**Ga5**–Ga3	109.63(4)	×2	Ga3–**Ga8**–Ga5	59.95(4)	×2
Ga1–**Ga5**–Ga5	58.50(4)	×2	Ga3–**Ga8**–Ga6	109.56(5)	×2
Ga1–**Ga5**–Ga7	59.84(5)	×2	Ga3–**Ga8**–Ga7	105.87(5)	×2
Ga1–**Ga5**–Ga8	112.90(6)	×2	Ga5–**Ga8**–Ga6	103.02(5)	×2
Ga3–**Ga5**–Ga5	58.23(4)	×2	Ga5–**Ga8**–Ga7	56.36(4)	×2
Ga3–**Ga5**–Ga7	115.27(5)	×2	Ga6–**Ga8**–Ga7	57.11(4)	×2
Ga3–**Ga5**–Ga8	61.31(5)	×2			
Ga5–**Ga5**–Ga7	106.34(7)	×2			
Ga5–**Ga5**–Ga8	106.03(7)	×2			
Ga7–**Ga5**–Ga8	66.78(5)	×2			

Table S6. Ga–Ga–Ga bond angles for the four-bonded Ga atoms of Na_2Ga_7.

**∡Ga–Ga–Ga**	**deg / °**		**∡Ga–Ga–Ga**	**deg / °**	
Ga1–**Ga9**–Ga3	130.36(9)	×1	Ga2–**Ga10**–Ga4	120.55(9)	×1
Ga1–**Ga9**–Ga8	98.95(3)	×2	Ga2–**Ga10**–Ga7	98.99(3)	×2
Ga3–**Ga9**–Ga8	105.41(3)	×2	Ga4–**Ga10**–Ga7	109.83(3)	×2
Ga8–**Ga9**–Ga8	119.28(5)	×1	Ga7–**Ga10**–Ga7	118.58(5)	×1

Table S7. Molar ratio of the reaction products from a thermal decomposition experiment of Na_7Ga_{13} after different reaction times *t*.

***t* / h**	**Composition / mol-%**		
	Na_7Ga_{13}	Na_2Ga_7	$NaGa_4$
0	100	0	0
0.25	87	13	0
0.5	74	26	0
1	46	54	0
2	12	88	0
4	0	87	13
6	0	34	66
12	0	7	93
24	0	0	100

Table S8a. Lattice parameters of Na_2Ga_7 after annealing at different temperatures refined from 52 non-overlapping reflections.

T / °C	200	300	450
a / Å	14.8550(8)	14.8555(8)	14.8546(7)
b / Å	8.6811(4)	8.6814(4)	8.6811(4)
c / Å	11.6092(9)	11.6084(9)	11.6073(8)
V / Å³	1497.1(2)	1497.1(2)	1496.8(1)

Table S8b. Lattice parameters of Na_2Ga_7 in the two-phase regions with $NaGa_4$ and Na_7Ga_{13}. Molar fractions of Na and annealing temperatures are given. Parameters were refined from the same subset of 20 non-overlapping reflections.

T / °C	200			300		
x(Na) / %	21	22.22	27	21	22.22	27
a / Å	14.854(3)	14.856(4)	14.856(3)	14.855(5)	14.855(1)	14.855(3)
b / Å	8.678(2)	8.682(3)	8.682(2)	8.683(4)	8.682(1)	8.683(3)
c / Å	11.612(2)	11.609(3)	11.608(2)	11.606(4)	11.611(1)	11.608(3)
V / Å³	1496.8(5)	1497.3(8)	1497.2(5)	1497(1)	1497.5(3)	1497.3(7)

Table S8c. Lattice parameters of Na_2Ga_7 from different preparation methods at 300 °C. Parameters were refined from the same subset of 20 non-overlapping reflections.

Method	From the elements	Na_7Ga_{13} + $NaGa_4$	$NaGa_4$ + $NaNH_2$	Thermal decomp. of Na_7Ga_{13}	Na_7Ga_{13} + NH_3
a / Å	14.855(1)	14.855(3)	14.858(3)	14.853(3)	14.858(3)
b / Å	8.682(1)	8.681(3)	8.684(3)	8.682(3)	8.684(3)
c / Å	11.611(1)	11.611(3)	11.607(3)	11.608(3)	11.611(3)
V / Å³	1497.5(2)	1497.3(7)	1497.6(7)	1496.9(7)	1498.1(7)

Table S9. Optimized atomic coordinates of Na_2Ga_7.

Atom	Site	*x*	*y*	*z*
Ga1	4*c*	–0.15216	–1/4	0.32711
Ga2	4*c*	–0.13562	1/4	0.18335
Ga3	4*c*	0.07286	–1/4	0.04840
Ga4	4*c*	–0.42259	1/4	0.04464
Ga5	8*d*	–0.19514	0.41072	–0.16200
Ga6	8*d*	–0.29443	0.09841	0.16775
Ga7	8*d*	–0.33374	0.49537	–0.04123
Ga8	8*d*	–0.15281	0.49353	0.04990
Ga9	4*c*	–0.08646	–1/4	0.12099
Ga10	4*c*	0.40910	1/4	0.10180
Na1	4*c*	–0.28485	–1/4	0.10938
Na2	4*c*	–0.10525	–1/4	–0.12981
Na3	8*d*	0.00923	–0.46172	0.31079

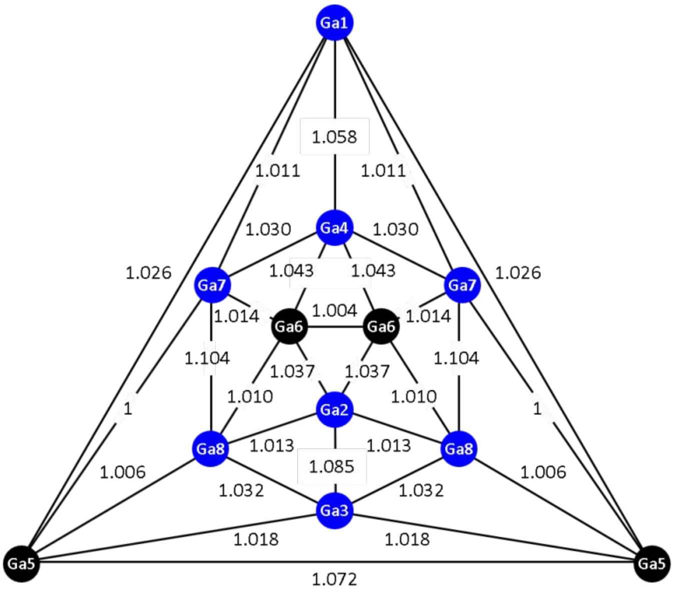

Figure S3. Schlegel diagram of the Ga_{12} icosahedron with distances normalized to the shortest Ga–Ga bond. Atoms connected with four-bonded Ga atoms are drawn in blue, atoms forming exohedral bonds to neighboring icosahedrons in black.

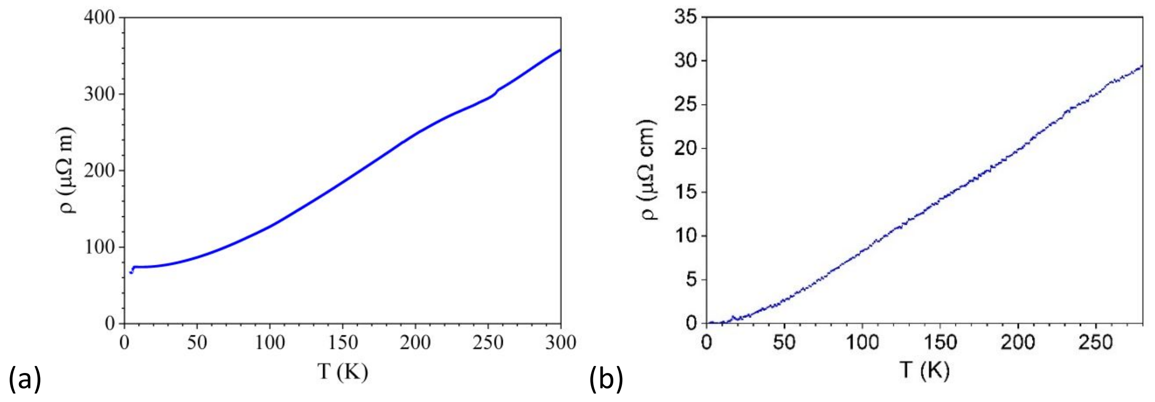

Figure S4. (a) Temperature dependence of electrical resistivity *ρ*(*T*) for a polycrystalline bulk sample of Na_2Ga_7 in the temperature range from *T* = 4 K to 300 K (–269.15 °C to 26.85 °C) in zero magnetic field. (b) Temperature dependence of electrical resistivity *ρ*(*T*) for elemental Ga in the temperature range from *T* = 2 K to 280 K (–275.15 °C to 6.85 °C).

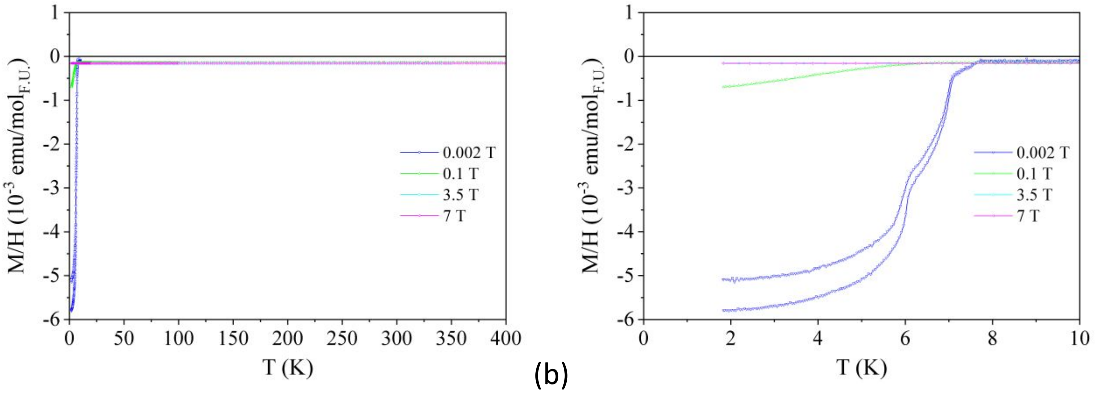

(a) (b)

Figure S5. (a) Temperature dependence of magnetic susceptibility χ(*T*) of Na_2Ga_7 in the temperature range from *T* = 1.8 K to 400 K (–271.35 °C to 126.85 °C) in magnetic fields $\mu_0 H$ = 0.002 T, 0.1 T, 3.5 T and 7 T. (b) Zoomed temperature dependence χ(*T*) for temperatures *T* = 1.8 K to 10 K (–271.35 °C to –263.15 °C).

References

(1) Müller, H.-P.; Hoppe, R. The Crystal Structure of $KGaO_2$, and $NaGaO_2$ (II). *Z. anorg. allg. Chem.* **1992**, *611*, 73−80.

(2) Chen, K.-C.; Fang, M.-H.; Huang, W.-T.; Kamiński, M.; Majewska, N.; Leśniewski, T.; Mahlik, S.; Leniec, G.; Kaczmarek, S. M.; Yang, C.-W.; Lu, K.-M.; Sheu, H.-S.; Liu, R.-S. Chemical and Mechanical Pressure-Induced Photoluminescence Tuning via Structural Evolution and Hydrostatic Pressure. *Chem. Mater.* **2021**, *33*, 3832−3840.
